# Improvement in Dibenzofuran-Based Hole Transport Materials for Flexible Perovskite Solar Cells

**DOI:** 10.3390/molecules29061208

**Published:** 2024-03-08

**Authors:** Yuanqiong Lin, Xiao Zhang, Jinchuan Lu, Xiaohan Lin, Yinghua Lu, Xin Li, Song Tu

**Affiliations:** 1 Pen-Tung Sah Institute of Micro-Nano Science and Technology, Xiamen University, Xiamen 361005, China; yuanqionglin@stu.xmu.edu.cn (Y.L.); zhangx9849@gmail.com (X.Z.); 2 College of Chemistry and Chemical Engineering, Xiamen University, Xiamen 361005, China; lujinchuan1536@gmail.com (J.L.); 20620211151972@stu.xmu.edu.cn (X.L.); ylu@xmu.edu.cn (Y.L.); 3 School of Electronic Science and Engineering, Xiamen University, Xiamen 361005, China

**Keywords:** hole transport materials, oligomers, dibenzofuran, π-conjugation modulation, perovskite solar cells

## Abstract

The π-conjugated system and the steric configuration of hole transport materials (HTMs) could greatly affect their various properties and the corresponding perovskite solar cells’ efficiencies. Here, a molecular engineering strategy of incorporating different amounts of p-methoxyaniline-substituted dibenzofurans as π bridge into HTMs was proposed to develop oligomer HTMs, named **mDBF**, **bDBF**, and **tDBF**. Upon extending the π-conjugation of HTMs, their HOMO energy levels were slightly deepened, significantly increasing the thermal stability and hole mobility. The incorporation of p-methoxyaniline bridges built one or two additional triphenylamine propeller structures, resulting in a denser film. Here, the **tDBF**-based n-i-p flexible perovskite solar cells createdchampion efficiency, giving a power conversion efficiency of 19.46%. And the simple synthesis and purification process of **tDBF** contributed to its low manufacturing cost in the laboratory. This work provided a reference for the development of low-cost and efficient HTMs.

## 1. Introduction

Perovskite solar cells (PSCs) are considered a promising candidate for solving current energy problems due to their excellent photoelectric conversion efficiency, low manufacturing cost, and adjustable optical band gap [1,2]. In just a few decades, the power conversion efficiency (PCE) of PSCs has surged from 3.8% in 2009 to 26.1% in 2023 [3,4,5], a growth rate that no other solar cell can match. As one of the key components, hole transport materials (HTMs) play a crucial role in extracting and transporting holes from the perovskite to the cathode. A large number of HTMs have been developed and have obtained good results in the field of PSCs (Table S1). The PCE of PSCs based on polymers such as poly[3-(4-carboxybutyl)thiophene-2,5-diyl] (P3CT) has exceeded 21% [6]. And 2,2′,7,7′-tetrakis (*N*,*N*-di-*p-*methoxyphenylamine)-9,9′-spirobifluorene (**Spiro-OMeTAD**) remains the benchmark for PSCs [7,8]. The use of deliquescent dopants in **Spiro-OMeTAD** enhances its hole mobility and conductivity while introducing device instability, the latter being undesired. Therefore, the rational design and development of efficient and low-cost hole transport materials is still necessary.

Oligomers combine good film-forming properties, defined structure, and easy purification, which are superior to small-molecule compounds and conjugated polymers [9,10]. Oligomers also display greater advantages in terms of forming specific condensed structures and precisely controlling supramolecular structures. Based on these, they are excellent candidates for studying the relationship between the structure and properties of different conjugated systems. The reported oligomeric HTMs all confirm these ideas, such as oligomer HTMs containing oligothiophene and triphenylamine [11], HTMs based on dangling methylsulfanyl-substituted fluorene units as repeating units [12], and the foldable HTMs with different amounts of methylene groups in carbazole bearing two 3,6-bis(4,4′-dimethoxydiphenylamino) [13]. In addition, the ideal π-conjugated system and the steric configuration of HTMs are crucial in improving their properties. Cheng et al. expanded π-conjugations by adding additional 4,4′-dimethoxy-diphenylamine units to HTM based on the spiro [fluoro-9,9′-oxanthracene] (SFX) core [14], thereby increasing the hole mobility of HTM. Their team also inserted benzene rings into 9,10-dihydroacridine (ACR)-based HTMs to obtain high glass transition temperatures [15], the highest of which reached 202 °C. Ding and his associates reported a modified Spiro-based material in which the four anisole units on the **Spiro-OMeTAD** are replaced by 4-methoxybiphenyl [16], which provides high hole extraction/transport capacity and excellent thermal properties due to π-conjunction extension. However, reports on linear dibenzofuran-based HTMs with different lengths of π-conjugated chains for flexible n-i-p PSCs are rare.

Herein, this work aimed to provide a comprehensive understanding of the effect of extending the specified π-conjugated skeleton on HTMs’ properties and the corresponding device performance with the design and synthesis of low-cost oligo-dibenzofuran-based HTMs (**mDBF**, **bDBF**, and **tDBF**). The photophysical and electrochemical properties of three HTMs were investigated using UV–vis absorption spectroscopy, fluorescence spectra, and cyclic voltammetry curves. The above results were verified and supplemented by density functional theory calculations (DFT). The excellent thermal properties of materials were confirmed based on thermogravimetric (TGA) and differential scanning calorimetry (DSC) results. And the film properties of the material, such as film morphology and hole transport rate, were also studied by scanning electron microscopy (SEM) and space charge limited current (SCLC) techniques. It was found that p-methoxyaniline bridges were introduced between the dibenzofuran units to construct several additional triphenylamine propeller structures to modulate the film morphology of HTMs. Furthermore, these three materials were, respectively, applied to flexible n-i-p PSCs as hole transport layers. The corresponding current density–voltage (*J-V*) curves and the external quantum efficiency (EQE) spectra were obtained to evaluate the device’s performance.

## 2. Result and Discussion

**Figure 1:**
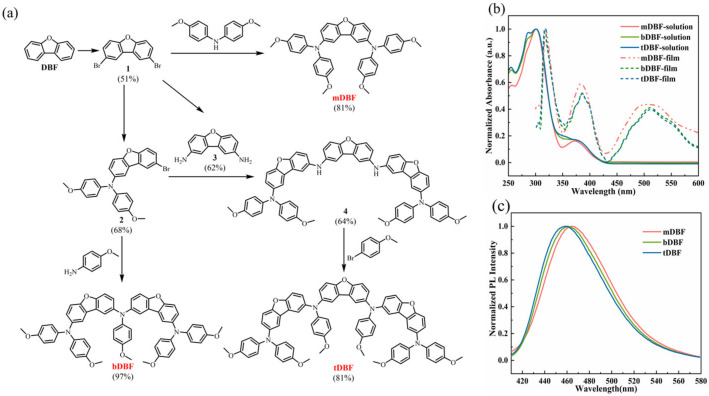
(**a**) Synthetic routes of the **mDBF**, **bDBF**, and **tDBF**; (**b**) UV–vis absorption spectra, (**c**) emission spectra of HTMs in dilute solution (dichloromethane, 10^−5^ M).

The development of oligomer HTMs has facilitated the learning of the structure–properties relationship of materials, which in turn, leads to a more accurate design of efficient and cost-effective HTMs. In this work, p-methoxyaniline-substituted dibenzofuran was introduced into oligomer HTMs due to the good planarity, flexible molecular structure design, and high thermal stability of dibenzofuran [17,18,19]. Bis(4-methoxyphenyl) amine was selected as an end group of the HTMs, which can accelerate hole transport because of its ability to enhance molecular coplanarity and intramolecular charge delocalization [20,21]. The resultant three HTMs had different properties, as evidenced by the large improvement in thermal stability and film morphology with the increase in the number of p-methoxyaniline-substituted dibenzofurans. And the corresponding device performance underwent similar changes. The specific synthetic routes of three HTMs are depicted in Figure 1a. Target HTMs could be synthesized by several simple reactions (bromination, amination, and the Buchwald–Hartwig coupling reaction), reducing the cost of synthesis. On the other hand, the selection of key intermediates (compound **2**) simplified the synthesis steps in the synthesis process, which further reduced the synthesis cost. According to the cost model proposed by Osedach et al. [22], the lab synthesis costs of **mDBF**, **bDBF**, and **tDBF** were estimated to be USD 5 g^−1^, USD 17 g^−1^, and USD 26 g^−1^, respectively. Therefore, new HTMs were more economical compared to **Spiro-OMeTAD**. All intermediates and final target products involved in the synthesis were confirmed by ^1^H NMR and ^13^C NMR, and even MALDI-TOF MS spectra, as shown in Figures S1–S16 (Supporting Materials).

To briefly investigate the effect of the molecular structure of HTMs on the conjugation effect, UV–vis absorption spectra of new HTMs in the dichloromethane solutions and thin films were recorded and are shown in Figure 1b. In dichloromethane solutions, the absorption curves of **mDBF**, **bDBF**, and **tDBF** displayed a very similar pattern with maximum absorption at 301 nm, which could be attributed to the π-π* transition of the molecular conjugation system [17,18]. For **mDBF**, another lower peak was observed at 372 nm, which was the result of intramolecular charge transfer from the core unit to the terminal group in **mDBF**. For both **bDBF** and **tDBF**, two shoulder peaks appeared at around 291 and 372 nm, showing that the extended π-conjugated chains led to stronger interchain interaction [23,24]. Even **mDBF**, **bDBF**, and **tDBF** did not have distinct absorption peaks in the visible region, indicating that there was no competition of light absorption between the designed HTMs and the perovskite light absorbing layer. In the thin film states, the absorption profiles of all three materials were redshifted, with their maximum absorption peaks shifted to 318 nm. In addition, all thin film absorption curves showed an additional absorption peak at about 511 nm. The above phenomena are suggestive of enhanced intermolecular interactions in the thin film state. Based on the absorption edges of **mDBF**, **bDBF**, and **tDBF** (Figure S17), the corresponding optical band gaps were calculated to be 2.17, 2.12, and 2.11 eV, respectively. Meanwhile, the emission spectra of **mDBF**, **bDBF**, and **tDBF** exhibited an emission band at 464, 461, and 459 nm. The strong emission character was highly conducive to converting UV light in solar irradiation into visible light, which could assist the perovskite absorber layer to accomplish more light absorption [25]. Based on the corresponding emission peaks (Figure 1c), large Stokes shifts of 163, 160, and 158 nm were calculated, demonstrating that a large structural change in the excited state occurred for three compounds [26].

**Figure 2:**
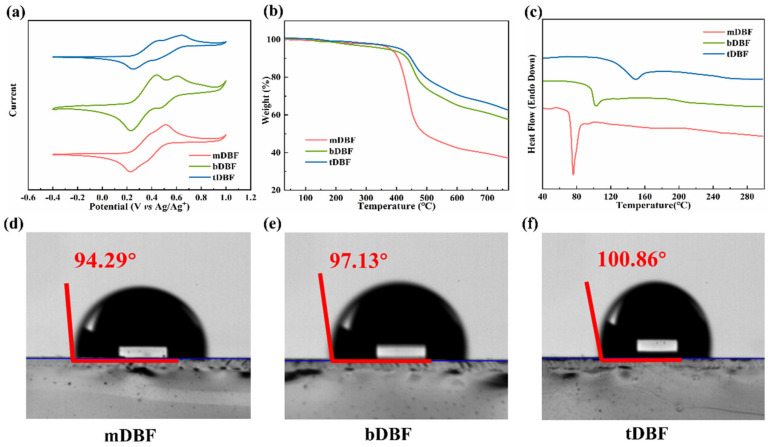
(**a**) Cyclic voltammogram of **mDBF**, **bDBF**, and **tDBF**; (**b**) TGA curves, (**c**) DSC curves of **mDBF**, **bDBF**, and **tDBF**; water contact angle test of (**d**) **mDBF**, (**e**) **bDBF**, and (**f**) **tDBF** coated on ITO.

The HOMO level and LUMO level of HTMs had an important impact on the devices’ performance. The appropriate energy level could promote the extraction of holes and prevent the injection of electrons, which in turn, reduces the recombination of holes and free electrons at the interface. Therefore, the HOMO and LUMO levels of **mDBF**, **bDBF**, and **tDBF** were measured by cyclic voltammetry, as shown in Figure 2a. The excellent electrochemical stability of the three HTMs was inferred from their highly reversible redox peak pairs [17,27]. According to the formulas $E_{HOMO}=-|E_{OX}-E_{(Fc/Fc+)OX}+5.11|$, the HOMO levels of **mDBF**, **bDBF**, and **tDBF** were found to be −5.27, −5.29, and −5.30 eV, respectively. Ulteriorly, the LUMO levels reckoned from the HOMO levels and $E_{g}^{opt}$ were −3.10, −3.17, and −3.19 eV, respectively. Based on the above experimental result, it could be presumed that increasing the number of p-methoxyaniline-substituted dibenzofurans units slightly lowered the HOMO and LUMO energy levels of the HTMs within a certain range.

**Figure 3:**
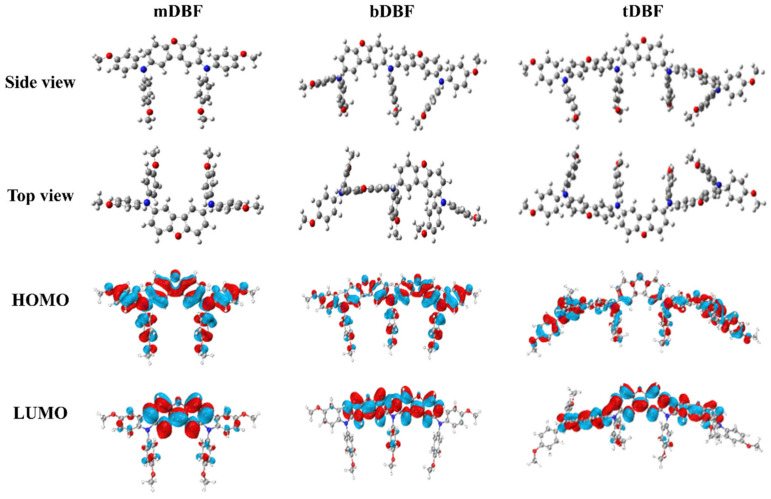
Results of DFT calculation (optimized structure, electron distributions in HOMO and LUMO energy levels) of **mDBF**, **bDBF**, and **tDBF**.

To gain more insight into the structure and electron cloud distribution of HTMs, a computational study of HTMs was carried out by quantum chemical theory calculations. The calculation program used for density functional theory (DFT) was Gaussian 09, and the methods and basis set are B3LYP and def2-SVP, respectively. The best molecular configurations (both side and top view) of **mDBF**, **bDBF**, and **tDBF** were obtained by DFT optimization simulations, as depicted in Figure 3. The optimized molecular spatial configuration showed that the dibenzofuran fragment maintained its original planarity in all molecules, which made for a better π-π stacking, and its benzene ring unit with bis(4-methoxyphenyl) amine and p-methoxyaniline separately constructed the propeller configuration of the triphenylamine group to modulate the film-forming ability of HTMs. Among them, **mDBF** had the best planarity, which was conducive to molecular stacking and intermolecular hole transport, but its film formation was also poor. In contrast, **bDBF** and **tDBF** had a certain torsion angle between dibenzofuran units due to the insertion of a p-anisidine bridge, resulting in better film morphology and a more compact interfacial contact between HTMs and the perovskite active layer. In other words, the risk of direct contact between the perovskite layer and the metal electrode was greatly reduced. On the other hand, it was known from the frontier molecular orbital model of DFT simulations that the HOMO of all HTMs was fully delocalized within the whole molecule backbone, which was beneficial for the overlap between its HOMO energy level and the valence band maximum of perovskite. In addition, the LUMO levels of all three HTMs mentioned above were shifted to the central dibenzofuran unit. Therefore, the electron distribution of HOMO and LUMO had a large overlap, which facilitated exciton generation and charge transport [18,28]. In addition, the number of dibenzofuran units (1~3) had an active effect on the energy levels of the molecules. As shown in Table 1, the trends of HOMO and LUMO energy levels obtained from DFT calculations agreed well with the experimental results and could be verified against each other.

The thermal stability of HTMs was also an important parameter to evaluate their durability and stability, so the thermal properties of these three materials were analyzed by thermogravimetric analysis (TGA) and differential scanning calorimetry (DSC) and further compared, as displayed in Figure 2b,c. It was found that the *T*_d_ of **mDBF**, **bDBF**, and **tDBF** was about 380, 372, and 418 °C, comparable to the thermal decomposition and/or phase transition temperatures of hybrid organic–inorganic perovskite crystals [20,29]. The results revealed that all three HTMs had excellent thermal stability and avoided decomposition behavior during the device fabrication process. Meanwhile, the insertion of more dibenzofuran units resulted in better thermal stability [30]. For the DSC curves, a melting peak was detected at 72 °C for **mDBF**. However, **bDBF** and **tDBF** all exhibited an obvious stepwise glassy shift during the heating process, with no obvious melting peak, indicating their amorphous structure. For **tDBF**, a higher *T*_g_ of 129 °C was observed, further illustrating its outstanding thermal stability and stable film morphology [31]. And this was another proof that an increase in the number of dibenzofuran units facilitated the improvement of the thermal stability of HTMs [32,33,34], which was conducive to the formation of a more stable amorphous structure.

In the n-i-p PSCs, HTMs also served to isolate the perovskite from moisture in the environment. To evaluate the hydrophobicity of HTMs, a water drop contact angles test was performed, as shown in Figure 2d–f. It was found that the undoped three HTMs had large water contact angles of 94.29, 97.13, and 100.86°, respectively. Among them, **tDBF** had the largest water contact angle and the best hydrophobicity, which might be due to the smallest proportion of methoxy groups in its structure [10]. It was concluded that the increase in p-methoxyaniline-substituted dibenzofuran units could improve the hydrophobicity and film-forming quality of HTMs, which in turn, was positive to the long-term stability of PSCs under unencapsulated conditions.

**Figure 4:**
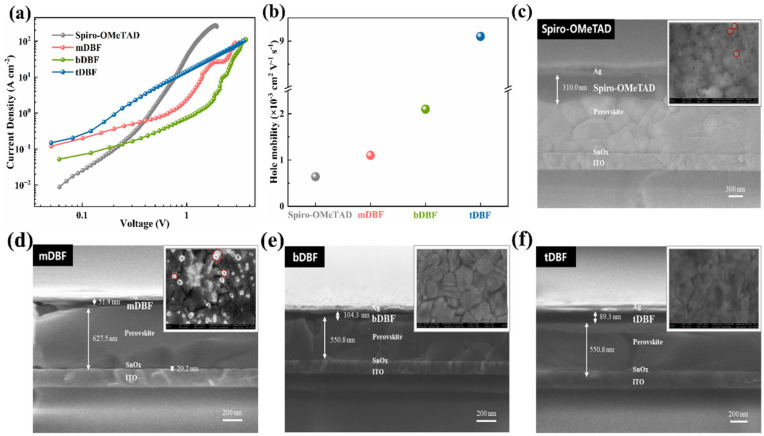
(**a**) *J-V* curves of the hole-only devices for **mDBF**, **bDBF**, and **tDBF**; (**b**) calculated hole mobility according to the Mott–Gurney law; (**c**) cross-sectional SEM image of PSCs with (**c**) **Spiro-OMeTAD**, (**d**) **mDBF**, (**e**) **bDBF**, and (**f**) **tDBF** hole transport layer. Inset: Top view SEM images of **mDBF**/perovskite, **bDBF**/perovskite, and **tDBF**/perovskite.

To assess the hole transport properties in the perovskite active layers with different HTMs, hole-only devices were fabricated with the structure of PET/ITO/NiO_x_/Cs_0.05_Rb_0.05_(FA_0.83_MA_0.17_)_0.90_Pb(I_0.95_Br_0.05_)_3_/HTMs/MoO_3_/Ag, where HTM was either **mDBF**, **bDBF**, and **tDBF** or **Spiro-OMeTAD**. And tBP and Li-TFSI with the optimized ratio participated in the preparation of the above-mentioned HTMs. Based on the space-charge-limited current (SCLC) method, the corresponding log *J-V* curves were derived to further calculate the hole mobility of the four HTMs, as exhibited in Figure 4a,b. The hole mobility values of **mDBF**, **bDBF**, and **tDBF** were 1.1 × 10^−3^, 2.9 × 10^−3^, and 9.1 × 10^−3^ cm^2^ V^−1^ s^−1^, respectively. This result further validated that hole transport ability was enhanced with the expansion of π-conjugation in HTMs. In addition, based on their better planarity and larger extension structure, they were endowed with higher hole mobility, which was one order of magnitude higher than that of doped **Spiro-OMeTAD** (6.4 × 10^−4^ cm^2^ V^−1^ s^−1^).

The planar n-i-p flexible PSCs were constructed to investigate the photovoltaic properties with **Spiro-OMeTAD**/**mDBF**/**bDBF**/**tDBF** as HTM, where the **Spiro-OMeTAD**-based device was the reference device. The cross-sectional view of the device scanned by SEM was depicted in Figure 4c–f. And all the layers with a device configuration of PET/ITO/SnO_x_/Cs_0.05_Rb_0.05_(FA_0.83_MA_0.17_)_0.90_Pb(I_0.95_Br_0.05_)_3_/HTM/MoO_3_/Ag could be clearly distinguished. It could be seen that the film thickness of **bDBF**, **tDBF**, and **Spiro-OMeTAD** was more uniform than that of **mDBF**, which indicated from the side that the film-forming ability of the first three was stronger. To further investigate the film morphology of three HTMs coated on perovskite, surface view SEM images of HTMs were recorded (inset of Figure 4c–f). The observed fact was that **tDBF** possessed the smoothest film without pinholes among the designed three HTMs. It was speculated that a larger torsion angle between adjacent dibenzofuran units reduced the planarity of the HTM and facilitated the formation of a solid film. Satisfyingly, the film-forming ability of **tDBF** even preceded that of **Spiro-OMeTAD**.

**Figure 5:**
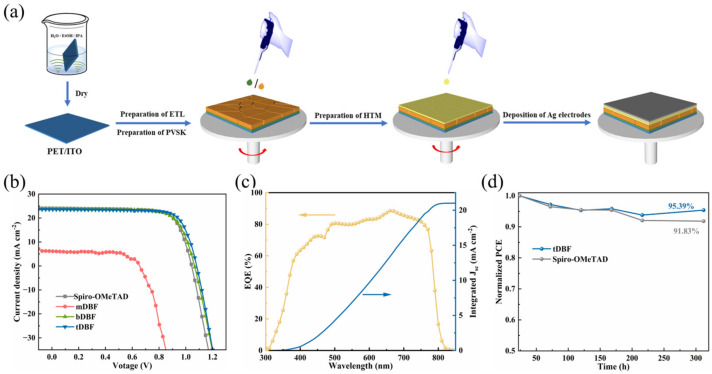
(**a**) Schematic diagram for device fabrication; (**b**) current density–voltage (*J*-*V*) curves of the PSCs based on different HTMs; (**c**) EQE spectra and integrated *J*_sc_ of the device based on **tDBF**; (**d**) normalized stability of **tDBF**- and **Spiro-OMeTAD**-based unencapsulated flexible devices in an air environment with 25 ± 5% relative humidity.

To further elucidate the influence of the developed HTMs with different amounts of p-methoxyaniline-substituted dibenzofurans on device efficiency, the current–voltage (*J-V*) characteristics of the corresponding PSCs were carried out. The processes for device fabrication and the corresponding *J-V* curves are demonstrated in Figure 5a,b, respectively. And the detailed photovoltaic parameters are summarized in Table S3. **mDBF**-based PSC delivered a short-circuit current density (*J*_sc_) of 6.11 mA cm^−2^, an open-circuit voltage (*V*_oc_) of 0.65 V, and a fill factor of (FF) 66.16%, ultimately resulting in an extremely low PCE of 2.65%. The device adopted **bDBF** as HTM gave a *J*_sc_ of 23.80 mA cm^−2^, *V*_oc_ of 1.07 V, and FF of 73.60%, achieving a PCE of 18.66%. The champion cell prepared with **tDBF** yielded a PCE of 19.46% with a *J*_sc_ of 23.54 mA cm^−2^, *V*_oc_ of 1.08 V, and FF of 76.47%. The slightly enhanced *V*_oc_ of the **bDBF**- and **tDBF**-based devices were considered to originate from their lower HOMO level compared to **mDBF**, and the slightly enhanced *J*_sc_ and FF were presumably due to the higher charge carrier mobility of **bDBF** and **tDBF** over **mDBF**. In addition, the role of better film formation caused by the insertion of more p-methoxyaniline-substituted dibenzofurans could not be ignored either. Finally, it was inspiring that **bDBF**- and **tDBF**-based device results were comparable to or better than those of PSCs based on **Spiro-OMeTAD** fabricated under similar conditions, where the latter displayed a PCE of 18.78% with a *J*_sc_ of 24.05 mA cm^−2^, *V*_oc_ of 1.04 V, and FF of 74.84%. The external quantum efficiency (EQE) spectra and corresponding integrated currents of the **tDBF** devices were measured and are provided in Figure 5c. EQE curves showed a wide range of optical responses, and the integrated *J*_sc_ was in good agreement with *J*_sc_ from the *J-V* measurement. This result further ensured the reliability of the *J-V* curves. The environmental stability of the unencapsulated flexible PSCs based on **tDBF** and **Spiro-OMeTAD** was also evaluated in the air with a relative humidity (RH) of around 25% at 25 °C, as displayed in Figure 5d. After 312 h, the **tDBF**-based flexible device retained a PCE of 95.39% of its initial value, while the **Spiro-OMeTAD**-based flexible device lost 8.17% of its original PCE. It could be seen that the **tDBF**-based device possessed slightly better environmental stability, which was consistent with its superior film-forming properties.

## 3. Materials and Methods

All raw materials and solvents involved in the synthesis process were provided by common commercial suppliers, including Bidepharm, Sinopharm, Adamas, Macklin, Energy Chemical, and Titan. It should be noted that toluene needed to be treated with calcium hydride before use. The rest of the materials could be used directly.

All high-purity chemicals used in the device fabrication process were purchased and were not subject to secondary processing before use. Cesium iodide (CsI, 99.9%), rubidium iodide (RbI, 99.9%), 4-tert-butylpyridine (tBP), lithium bis(trifluoromethylsulphonyl)imide (Li-TFSI, 520 mg mL^−1^, in acetonitrile) and the organic solvents (*N*,*N*-dimethylformamide (DMF, 99.8%), dimethyl sulfoxide (DMSO, 99.8%), and chlorobenzene (CB, 99.8%)) were originated from Sigma-Aldrich (St. Louis, MO, USA). The SnO_2_ precursor (15% in H_2_O) was purchased from Alfa Aesar (Hisham, UK). Lead iodide (PbI_2_, 99.99%) was gained from TCI (Tokyo, Japan). Formamidinium iodide (FAI) and methylammonium bromide (MABr) were sourced from Greatcell Solar Materials (New South Wales, Australia). PEN/ITO substrates (14 ohm square^−1^) were bought from Peccell (Tokyo, Japan). In addition, **Spiro-OMeTAD** was chosen as the reference HTM, which was provided by Derthon Optoelectronic Materials Science Technology (Shenzhen, China).

### 3.1. Synthesis of **mDBF**, **bDBF** and **tDBF**

Synthesis of 2,8-dibromodibenzo[b,d]furan (**1**): Dibenzofuran (1.00 g, 5.95 mmol) was uniformly dissolved in trichloromethane (6 mL), and Br_2_ (0.68 mL, 13.26 mmol) was added slowly dropwise under ice bath. The reaction was then stirred at room temperature for 26.5 h. Subsequently, Na_2_S_2_O_3_ aqueous solution (0.25 mol L^−1^, 40 mL) was added to the reaction mixture to terminate the reaction. Then extraction was performed, and the crude product was obtained by collecting the organic phase and removing the solvent. Finally, the crude product was recrystallized with toluene to obtain **1** (white needle-like crystals, 989 mg, 51.0%). ^1^H NMR (500 MHz, CDCl_3_, ppm): δ 7.44 (d, *J* = 8.5 Hz, 2H), 7.57 (dd, *J*_1_ = 8.5 Hz, *J*_2_ = 2.0 Hz, 2H), 8.02 (d, *J* = 2.0 Hz, 2H); 13C NMR (100 MHz; CDCl_3_, ppm) δ 113.40, 115.95, 123.71, 125.16, 130.75, 155.34.

Synthesis of *N*^2^, *N*^2^, *N*^8^, *N*^8^-tetrakis(4-methoxyphenyl)dibenzo[b,d]furan-2,8-diamine (**mDBF**): To a 25 mL two-necked round-bottom flask, **1** (0.49 g, 1.5 mmol), Bis-(4-methoxyphenyl)-amine (0.76 g, 3.3 mmol), Tris(dibenzylideneacetone)dipalladium(0) (Pd_2_(dba)_3_, 0.05 g, 0.06 mmol), 4,5-Bis(diphenylphosphino)-9,9-dimethylxanthene (Xantphos, 0.07 g, 0.12 mmol) and sodium tert-butoxide (0.43 g, 4.5 mmol) were added, followed by evacuation and argon backfilling. Then, anhydrous toluene (15 mL) was added to the above mixture and heated to reflux. After the reaction for 8 h, the resulting mixture was extracted three times with ethyl acetate (EA). The organic phase was collected and the solvent was removed to obtain the crude product. Finally, the crude product was purified by column chromatography to obtain **mDBF** (yellow powder, 700 mg, 81.0%). ^1^H NMR (500 MHz, CDCl_3_, ppm) δ 3.78 (s, 12H), 6.78~6.80 (m, 8H), 6.98~6.99 (m, 8H), 7.11~7.12 (m, 2H), 7.35~7.37 (m, 2H), 7.42 (s, 2H); ^13^C NMR (125 MHz, CDCl_3_, ppm): δ 55.51, 112.07, 114.67, 115.44, 123.95. 125.02, 125.07, 142.15, 144.22, 152.65, 155.12. C_40_H_34_N_2_O_5_ Exact Mass (622. 247), MS (MALDI-TOF) (622.125).

Synthesis of 8-bromo-*N*,*N*-bis(4-methoxyphenyl)dibenzo[b,d]furan-2-amine (**2**): This compound was synthesized according to the same procedure described for **mDBF**. The materials required and their dosages were as followe:**1** (2.01 g, 6.2 mmol), Bis-(4-methoxyphenyl)-amine (0.71 g, 3.1 mmol), Pd_2_(dba)_3_ (0.85 g, 0.09 mmol), Xantphos (0.13 g, 0.22 mmol), sodium tert-butoxide (0.60 g, 6.2 mmol) and anhydrous toluene (48 mL) were used. The crude product was purified by column chromatography using PE and EA as eluent (40:1) to afford **2** (bright yellow powder 996 mg, 67.7%). ^1^H NMR (400 MHz, CDCl_3_, ppm) δ 3.80 (s, 6H), 6.81~6.83 (m, 4H), 7.02~7.04 (m, 4H), 7.12~7.18 (m, 1H), 7.37~7.39 (m, 2H), 7.47~7.50 (m, 2H), 7.89~7.90 (m, 1H); ^13^C NMR (125 MHz, CDCl_3_, ppm): δ 55.53, 112.16, 113.16, 114.33, 114.76, 115.30, 123.60, 123.90, 125.53, 126.34, 129.76, 141.87, 144.94, 152.13, 155.45, 155.54.

Synthesis of *N*^2^-(8-(bis(4-methoxyphenyl)amino)dibenzo[b,d]furan-2-yl)-*N*^2^,*N*^8^,*N*^8^-tris(4-methoxyphenyl)dibenzo[b,d]furan-2,8-diamine (**bDBF**): The synthesis process of this compound differed from that of **mDBF** only in terms of materials and dosages. The specific materials and their dosages were as follows: **2** (1.42 g, 3 mmol), p-Anisidine (0.12 g, 1 mmol), Pd_2_(dba)_3_ (0.18 g, 0.2 mmol), Tri-tert-butylphosphine tetrafluoroborate (P(t-Bu)_3_ ‧HBF_4_, 0.17 g, 0.6 mmol), sodium tert-butoxide (0.48 g, 5 mmol) and anhydrous toluene (30 mL). Subsequently, the reaction mixture was heated and stirred at 110 °C for 8 h. Finally, the target product (**bDBF**) was obtained after purification by column chromatography (brownish-yellow powder, 886 mg, 97.4%). ^1^H NMR (500 MHz, CDCl_3_, ppm) δ 3.77 (s, 15H), 6.77~6.80 (m, 10H), 6.96~7.01 (m, 10H), 7.10~7.15 (m, 4H), 7.35~7.43 (m, 8H); ^13^C NMR (125 MHz, CDCl_3_, ppm): δ 55.52, 112.07, 114.65, 114.77, 115.25, 123.87. 124.98, 125.13, 125.58, 142.14, 144.17, 152.63, 155.13. C_59_H_47_N_3_O_7_ Exact Mass (909.341), MS (MALDI-TOF) (909.425).

Synthesis of dibenzo[b,d]furan-2,8-diamine (**3**): **1** (0.325 g, 1 mmol) and CuI (0.76 g, 0.40 mmol) were weighed and mixed in a Schlenk tube and then purged 3–5 times with argon. *N*,*N*′-dimethylethylenediamine (DMEDA, 64 μL, 0.60 mmol), 25–28% ammonia (1.5 mL), and DMSO (1.0 mL) were added to the above Schlenk tube. Subsequently, the mixture was heated and stirred at 130 °C for 27.5 h. After the reaction was completed and cooled to room temperature, saturated aqueous Na_2_SO_4_ solution (10 mL) was added. Then, the crude product was purified by extraction and column chromatography to obtain **3** (orange powder,124 mg, 62.4%). ^1^H NMR (500 MHz, CDCl_3_, ppm) δ 3.60 (s, 4H), 6.72 (dd, *J*_1_ = 9.0 Hz. *J*_2_ = 2 Hz, 2H), 7.07 (d, *J* = 2.0 Hz, 2H), 7.23 (d, *J* = 9.0 Hz, 2H).

Synthesis of *N*^2^, *N*^2^′-(dibenzo[b,d]furan-2,8-diyl)bis(*N*^8^, *N*^8^-bis(4-methoxyphenyl)dibenzo[b,d]furan-2,8-diamine) (**4**): This compound **4** was synthesized according to the same procedure described for **mDBF**. The materials involved and their dosages are as follows: **2** (0.71 g, 1.5 mmol), **3** (0.10 g, 0.5 mmol), Pd_2_(dba)_3_ (0.05 g, 0.05 mmol), (S)-2,2′-bis(diphenylphosphino)-1,1′-binaphthyl ((S)-BINAP, 0.03 g, 0.10 mmol), sodium tert-butoxide (0.14 g, 1.5 mmol), and anhydrous toluene (12.5 mL). Subsequently, the reaction was heated and stirred at 110 °C for 6 h. After extraction and desolvation, the residue was purified by column chromatography (PE: EA = 5:1) to obtain **4** (yellow powder, 316 mg, 64.2%). ^1^H NMR (400 MHz, CDCl_3_, ppm) δ 3.74 (s, 12H), 5.63 (s, 2H), 6.76~6.78 (m, 8H), 6.97~6.99 (m, 8H), 7.08~7.11 (m, 6H), 7.34~7.46 (m, 12H); ^13^C NMR (125 MHz, CDCl_3_, ppm): δ 55.51, 109.78, 109.96; 112.08, 112.22, 112.25, 114.67, 115.21, 119.15, 119.29, 123.86, 124.99, 125.07, 125.10, 125.13, 139.82, 139.96, 142.13, 144.21, 152.30, 152.37, 152.61, 155.14.

Synthesis of *N*^2^, *N*^2^′-(dibenzo[b,d]furan-2,8-diyl)bis(*N*^2^, *N*^8^, *N*^8^-tris(4-methoxyphenyl)dibenzo[b,d]furan-2,8-diamine) (**tDBF**): **tDBF** was synthesized according to the same procedure described for **mDBF**, where **4** (0.59 g, 0.6 mmol), p-methoxybromobenzene (0.56 g, 3.00 mmol), Pd_2_(dba)_3_ (0.11 g, 0.12 mmol), P(t-Bu)_3_ ‧HBF_4_ (0.07 g, 0.24 mmol), sodium tert-butoxide (0.29 g, 3.00 mmol), and anhydrous toluene (18 mL) were used. Additionally, the mixture was heated and stirred at 110 °C for 10 h. The crude product was purified by column chromatography using PE and EA as eluent (3:1) to obtain **tDBF** (yellow-brown powder, 608 mg, 84.6%). ^1^H NMR (500 MHz, CDCl_3_, ppm) δ 3.76 (s, 18H). 6.76~6.78 (m, 12H), 6.94~6.98 (m, 12H), 7.09~7.13 (m, 6H), 7.34~7.40 (m, 12H); ^13^C NMR (125 MHz, CDCl_3_, ppm): δ 55.50, 112.04, 112.19, 114.64, 114.76. 115.72, 115.81, 123.88, 124.07, 124.14, 125.09, 125.27, 142.10, 142.21, 144.30, 144.37, 152.74, 155.09, 155.22. C_78_H_60_N_4_O_9_ Exact Mass (1196.436), MS (MALDI-TOF) (1196.527).

### 3.2. Characterization and Analysis Methods

^1^H NMR and ^13^C NMR spectra were obtained using a Bruker 400 MHz and 500 MHz spectrometer using tetramethylsilane (TMS) as the reference material. Time-of-flight mass spectrometer (MALDI-TOF-MS) experiments were performed by an MS Bruker autoflex maX (Bruker Corporation, Billerica, MA, USA) using two matrices, where, 2,5-dihydroxybenzoic acid (DHB) was the tested matrix for **mDBF**, while the tested matrix for **bDBF** and **tDBF** was trans-2-[3-(4-tert-butylphenyl)-2-methyl-2-propenylidene]-propanedinitrile (DCTB). Ultraviolet–visible absorption spectroscopy (UV–Vis) was recorded on a Cary 5000 UV–Vis-NIR spectrometer (Agilent Technologies, Ltd, Santa Clara, CA, USA). All solution samples were obtained by diluting a dichloromethane solution of the materials; film samples were prepared by spin coating a chlorobenzene solution of the material onto PET/ITO. Fluorescence spectra were obtained using a Hitachi F-7000 fluorescence spectrophotometer (Hitachi High-Tech, Tokyo, Japan). Cyclic voltammetry (CV) curves were tested using a Tatsuwa CHI6001 electrochemical workstation (Shanghai chenhua instrument co., ltd, Shanghai, China) with a scan rate of 50 mV s^−1^. The tests were performed under an argon atmosphere with a three-electrode system consisting of a platinum sheet as the counter electrode, a glassy carbon electrode as the working electrode, and an Ag/AgNO_3_ electrode (0.1 M AgNO_3_ in acetonitrile solution) as the reference electrode. The electrolyte was a dichloromethane solution of 0.1 M tetrabutylammonium hexafluorophosphate, and the measured cyclic voltametric results of ferrocene (Fc/Fc^+^) were used as an external calibration. According to the equations $E_{HOMO}=-|E_{OX}-E_{(Fc/Fc+)OX}+5.11|$ and $E_{LUMO}=E_{HOMO}+E_{g}^{opt}$, HOMO and LUMO energy levels were calculated, respectively. Moreover, dichloromethane was chosen as the test solvent for MALDI-TOF-MS, UV–Vis, and CV. Thermogravimetric (TGA) curves were obtained using an SDT 650+Discovery simultaneous thermal analyzer (TA Instruments, Newcastle, DE, USA), warming to 800 °C in an N_2_ atmosphere at a heating rate of 10 °C min^−1^. Differential scanning calorimetry (DSC) was employed using a TA DSC250 Instrument (TA Instruments, Newcastle, DE, USA) at a heating rate of 10 °C min^−1^ in a temperature range of 30 °C to 300 °C under a nitrogen atmosphere. The space-charge-limited current (SCLC) measurement was carried out using a Keithley 2420 system (Keithley Instruments, Inc, Cleveland, OH, USA) in the dark. The water contact angle of HTM was also tested using a DSA100 (KRUSS, Hamburg, Germany) contact angle/surface tension meter to evaluate its ability to protect perovskite from water erosion.

### 3.3. Device Fabrication

Pre-treatment of ITO conductive substrates: PET/ITO substrates that had undergone chemical etching were successively sonicated in deionized water, ethanol, and isopropanol for a quarter of an hour and then dried. Additionally, the resulting substrates were subjected to UV-ozone treatment for 0.5 h before the preparation of the SnO_2_ layer.

Preparation of the SnO_2_ layer: the purchased SnO_2_ precursor was further prepared to a diluted solution (2.14 wt%), followed by mixing with 5 mol% HCl and filtering for backup. Ulteriorly, the treated solution was sonicated for a quarter of an hour and filtered to obtain a homogeneous phase. Finally, the treated SnO_2_ precursor was spin-coated at 3000 rpm for 30 s and then heated at 120 °C for 0.5 h.

Preparation of perovskite films: the perovskite precursor solution was first prepared by mixing 167.03 mg FAI, 22.28 mg MABr, 599.31 mg PbI_2_, 47.8 μL CsI solution (1.5 M in DMSO), and 46.9 μL RbI solution (1.5 M in DMSO) in a mixed solvent (DMF/DMSO,743 μL/186 μL) and stirred overnight in a nitrogen glove box before use. Then, perovskite precursors were spin-coated onto the UV-ozonated substrates at 800 rpm for 10 s and 4000 rpm for 30 s, in that order. During the last 3 s of the high-speed period, 200 μL CB was added dropwise, and the resulting substrate was quickly transferred to a heating table for annealing at 120 °C for 0.5 h.

Preparation of HTMs: the synthesized HTM (**mDBF**, **bDBF**, and **tDBF**) was dissolved in CB at a concentration of 2.95 × 10^−5^ mol mL^−1^. Then, 7.97μL tBP and 4.84 μL of the stock solution of Li-TFSI (520 mg mL^−1^, in acetonitrile) were added to the above solution (553 μL). Finally, the obtained mixture was spin-coated onto the perovskite films at 2000 rpm for 30 s. Additionally, **Spiro-OMeTAD** was selected as the reference HTM, where 72.3 mg **Spiro-OMeTAD**, 17.5 μL of the stock solution of Li-TFSI, and 28.8 μL tBP were mixed in 1 mL CB.

Deposition of Ag electrodes: 3.6 nm of MoO_3_ and 100 nm of silver were vaporized onto the HTM layer under a high vacuum.

### 3.4. Device Performance Measurements

The HTM film morphology and microscopic morphologies of the typical device cross-sections were recorded on a scanning electron microscope (SEM, SUPRA 55 Zeiss, Oberkochen, Germany); the current density–voltage (*J-V*) curves of the PSCs were conducted under simulated AM 1.5 illumination (100 mW cm^−2^) using a Keithley 2400 source meter; the external quantum efficiency (EQE) spectra were obtained on a QE-R-3010 system. The hole mobility of the HTMs films was obtained by the space charge limited current (SCLC) technique. Here, PET/ITO/NiO_x_/Cs_0.05_Rb_0.05_(FA_0.83_MA_0.17_)_0.90_Pb(I_0.95_Br_0.05_)_3_/HTMs/MoO_3_/Ag was selected as a hole-only devices structure. And the log *J-V* curve was derived from the SCLC measure, and then the hole mobility was estimated according to the Mott–Gurney law:

(1) $$J=9\varepsilon_{r}\varepsilon_{0}\mu V^{2}/{(8L}^{3})$$

where *J* meant the current density; 𝜀_𝑟_ and 𝜀_0_ were corresponded to the organic material dielectric constant (𝜀_𝑟_ = 3) and the vacuum permittivity (𝜀_0_ = 8.85 × 10^−14^ F cm^−1^), respectively; 𝜇 was the hole mobility; *L* represented the thickness of the active layer, and *V* was the applied voltage.

## 4. Conclusions

In summary, a series of oligomer HTMs (**mDBF**, **bDBF**, and **tDBF**) based on p-methoxyaniline-substituted dibenzofurans were designed and synthesized to systematically investigate the effect of a π-conjugated system on the various properties of HTMs and the corresponding device efficiency. As the number of p-methoxyaniline-substituted dibenzofurans increased, the thermal stability, film morphology, and hydrophobicity of HTMs were improved as expected. The excellent thermal stability of dibenzofuran, the additional triphenylamine propeller structure constructed from p-methoxyaniline and dibenzofuran, and the increasing molecular weight made great contributions to the improved properties of HTMs. The extended π-conjugation also led to higher hole mobility of HTMs, where doped **tDBF** had the highest hole mobility of 9.1 × 10^−3^ cm^2^ V^−1^ s^−1^. In response to these, there was a clear increasing trend for the device efficiency of PSCs with **mDBF**, **bDBF**, and **tDBF** as HTMs. And **tDBF** was successfully applied as HTMs in n-i-p flexible PSCs, with the highest PCE of 19.46%. Meanwhile, the **tDBF**-based flexible device was more stable than the **Spiro-OMeTAD**-based flexible device in an air environment with 25 ± 5% RH, keeping 95.39% of its initial performance after 312 h. Encouragingly, **tDBF** was obtained by several classic chemical reactions with material costs of around USD 26 g^−1^, which were much cheaper than **Spiro-OMeTAD**. The results of this work highlighted precise molecular regulation by extending an appropriate π-conjugated system could have a dramatic impact on the properties of HTMs and device efficiency, paving the way for the development of novel low-cost and efficient HTMs.

## Figures and Tables

**Table 1:** Photophysical, electrochemical, and thermal properties of the molecules.

HTM	Experiment Data							Calculation Data ^e^	
	*λ*_max_ ^a^ (nm)	*λ*_stokes_ ^a^ (nm)	*E*_g_ ^b^ (eV)	*T*_d_ (°C)	*T*_g_ (°C)	HOMO ^c^ (eV)	LUMO ^d^ (eV)	HOMO (eV)	LUMO (eV)
**mDBF**	301	464	2.17	380	-	−5.27	−3.10	−4.64	−1.14
**bDBF**	301	461	2.12	372	98	−5.29	−3.17	−4.69	−1.27
**tDBF**	301	459	2.11	418	129	−5.30	−3.19	−4.71	−1.35

^a^ Measured in CHCl_2_ solution; ^b^ $E_{g}^{opt}=1240/\lambda_{edge}$ (absorption onset of the materials in thin film); ^c^ $E_{HOMO}=-\left|E_{ox}-E_{\left(Fc/{Fc}^{+}\right)ox}+5.11\right|$; ^d^ $E_{LUMO}=E_{HOMO}+E_{g}$; ^e^ Gaussian 09 at B3LYP and def2-SVP level.

## Data Availability

The data presented in this study are available in this article.

## Supplementary Materials

The following supporting information can be downloaded at: https://www.mdpi.com/article/10.3390/molecules29061208/s1, Figure S1 ^1^H NMR spectrum of **1**. Figure S2 ^13^C NMR spectrum of **1**. Figure S3 ^1^H NMR spectrum of **mDBF**. Figure S4. ^13^C NMR spectrum of **mDBF**. Figure S5. MALDI-TOF mass spectrometry of **mDBF**. Figure S6 ^1^H NMR spectrum of **2**. Figure S7 ^13^C NMR spectrum of **2**. Figure S8 ^1^H NMR spectrum of **bDBF**. Figure S9 ^13^C NMR spectrum of **bDBF**. Figure S10 MALDI-TOF mass spectrometry of **bDBF**. Figure S11 ^1^H NMR spectrum of **3**. Figure S12 ^1^H NMR spectrum of **4**. Figure S13 ^13^C NMR spectrum of **4**. Figure S14 ^1^H NMR spectrum of **tDBF**. Figure S15 ^13^C NMR spectrum of **tDBF**. Figure S16. MALDI-TOF mass spectrometry of **tDBF**. Figure S17. Tauc curves from UV-visible absorption spectrum of the materials in the thin films. Table S1 The relevant parameters obtained from PSCs with different HTMs. Table S2. Materials quantities and cost for the synthesis of 1-g **mDBF**, **bDBF** and **tDBF**. Table S3 Summary of photovoltaic parameters of n-i-p flexible PSCs employing different HTMs. References [35,36,37,38,39,40] are cited in the supplementary materials.
