# Supplementary material for: Improvement in Dibenzofuran-Based Hole Transport Materials for Flexible Perovskite Solar Cells
Source: Molecules. 2024 Mar 8;29(6):1208. doi: 10.3390/molecules29061208 (PMC10974200; doi:10.3390/molecules29061208)
Supplement: Supplementary file 1 [file molecules-29-01208-s001.zip › molecules-2889721-supplementary.pdf]

Supporting Materials

1. Structural characterization of intermediate compounds and HTMs

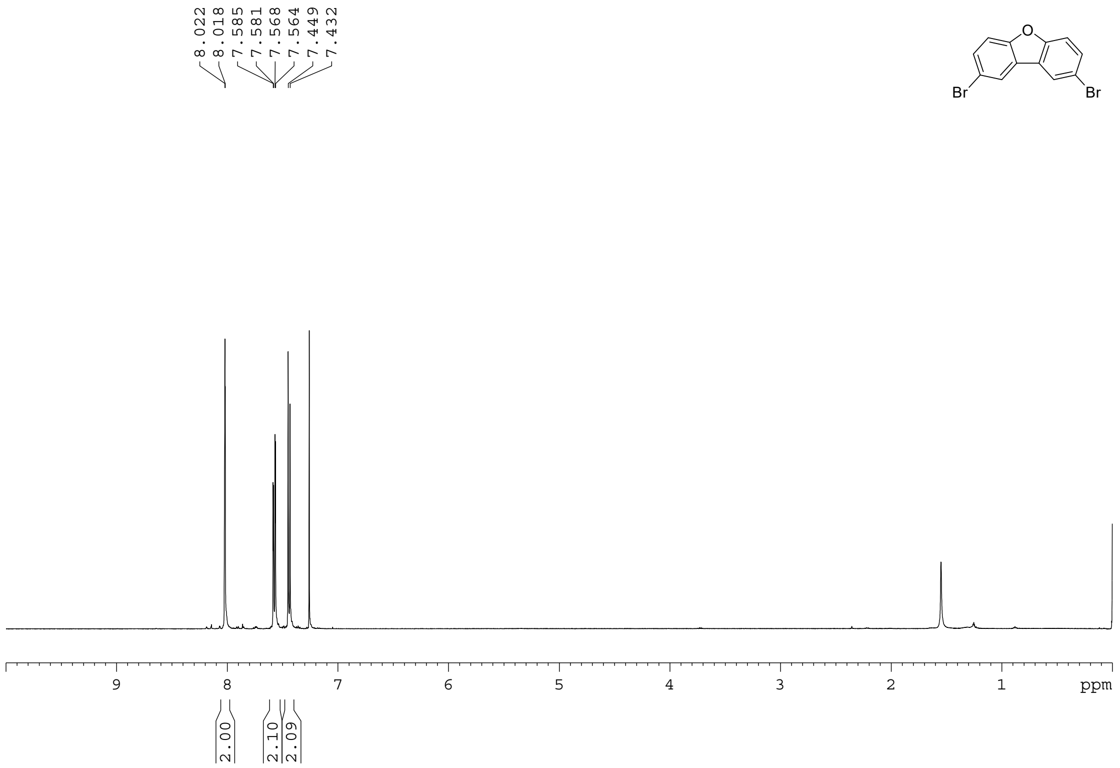

Figure. S1 ^{1}H NMR spectrum of **1**.

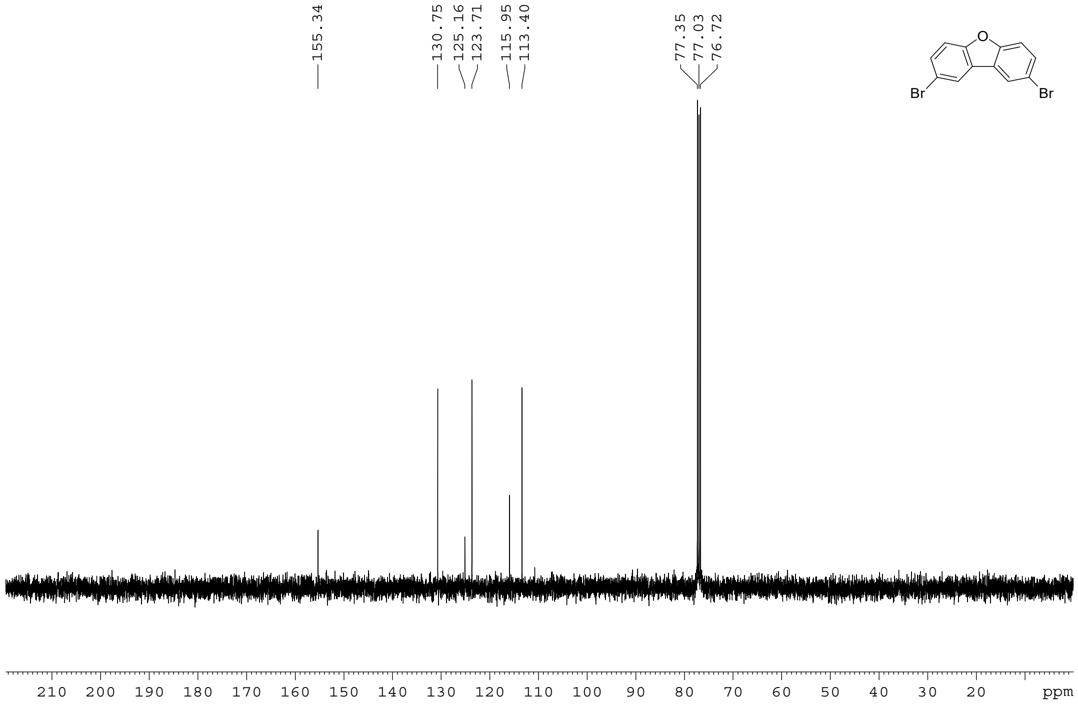

Figure. S2 ^{13}C NMR spectrum of **1**.

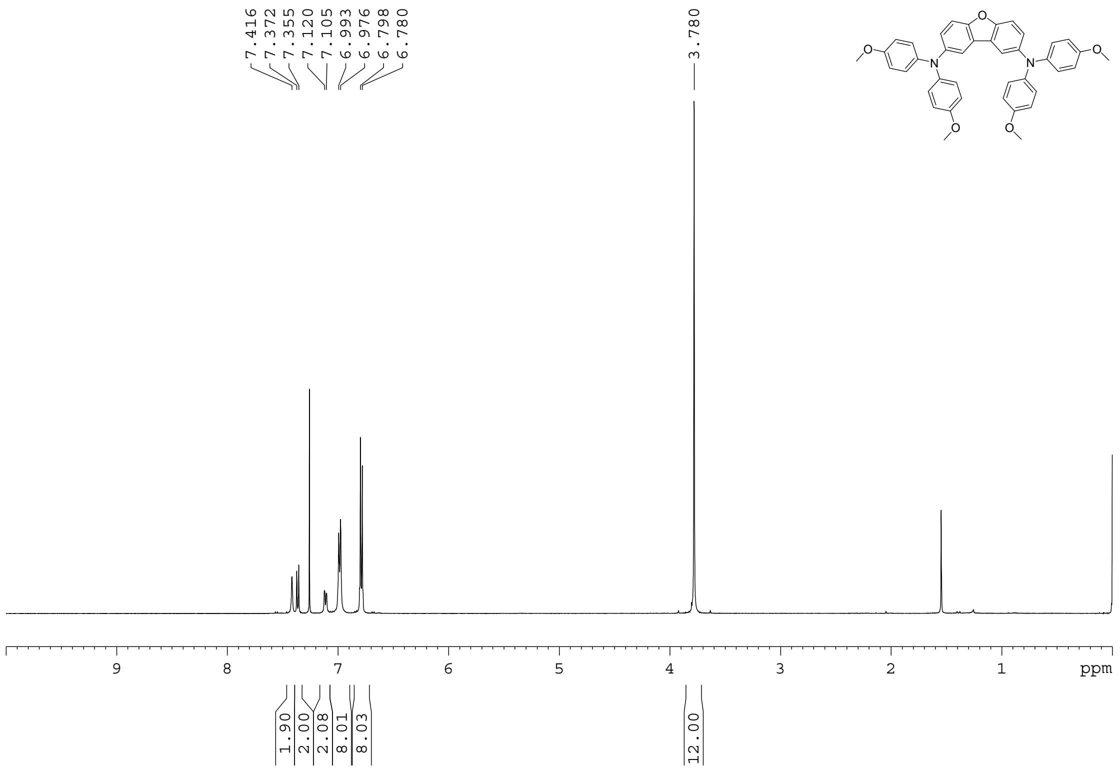

Figure. S3 ^{1}H NMR spectrum of **mDBF**.

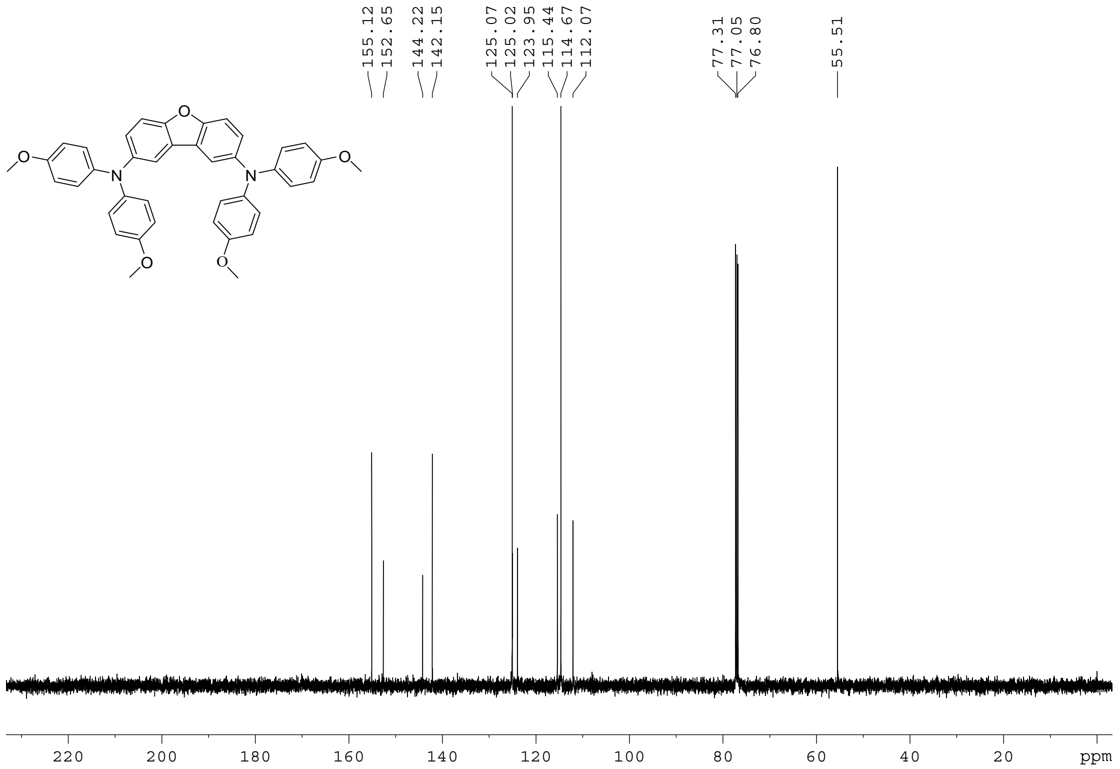

Figure. S4. ^{13}C NMR spectrum of **mDBF**.

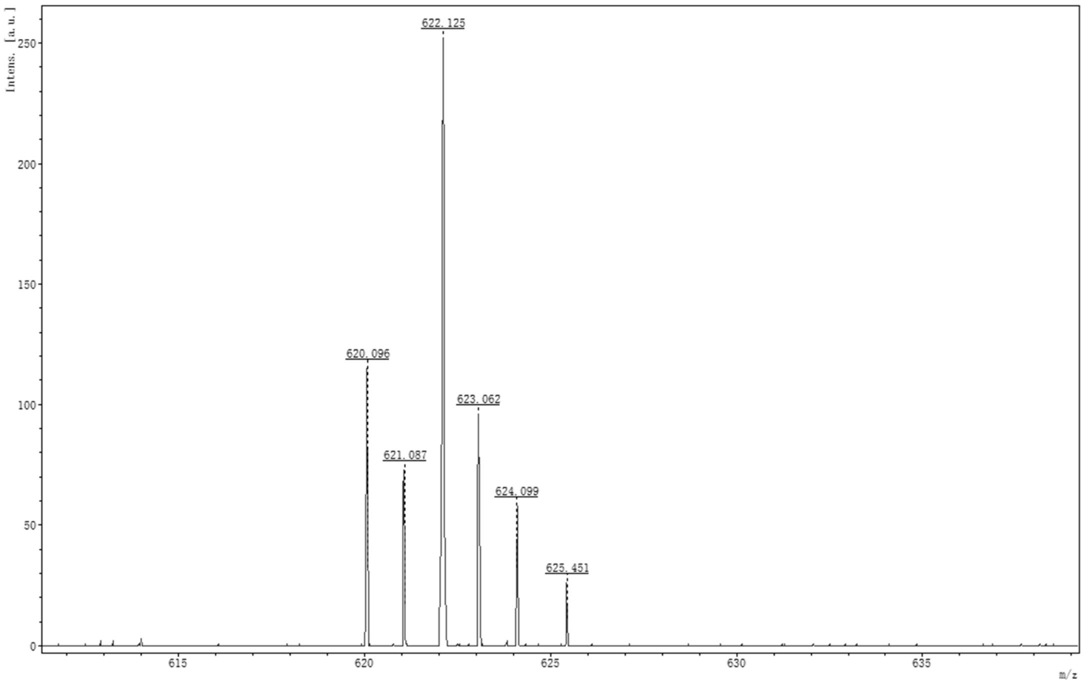

Figure. S5. MALDI-TOF mass spectrometry of **mDBF**.

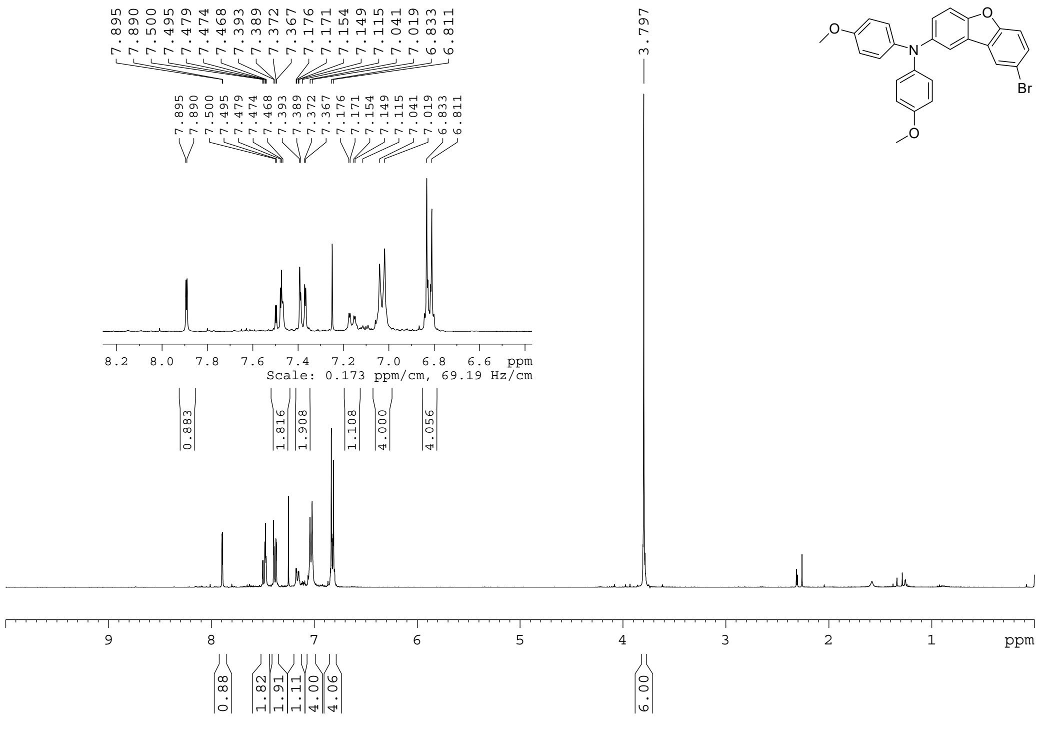

Figure. S6 ^{1}H NMR spectrum of **2**.

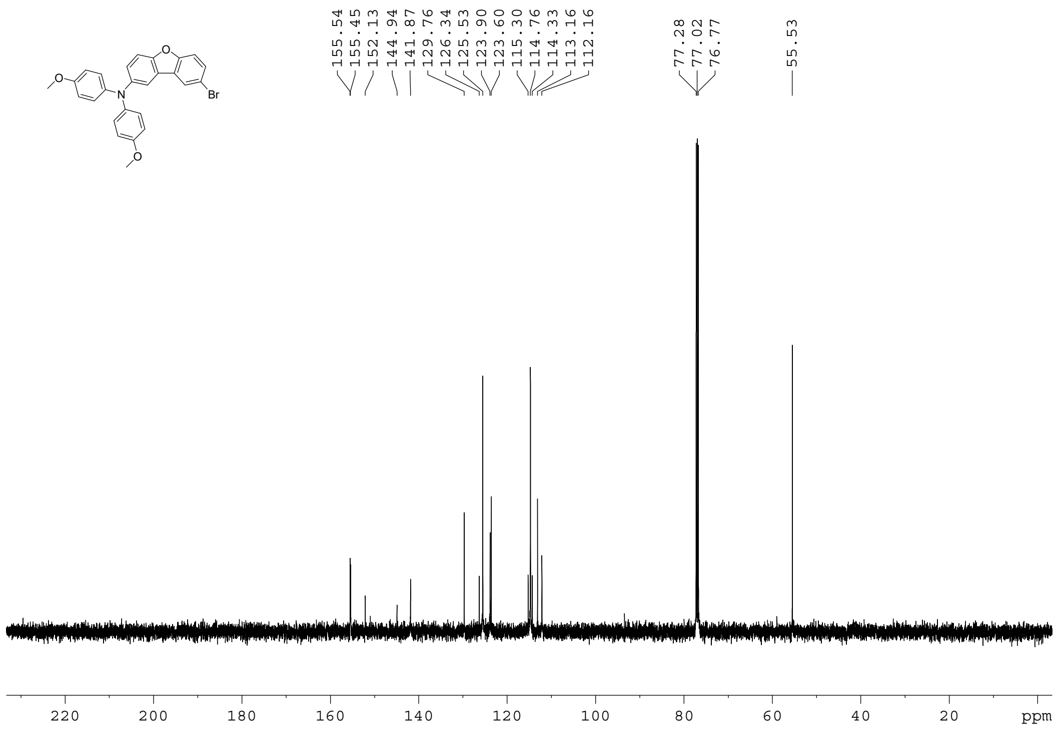

Figure. S7 ^{13}C NMR spectrum of **2**.

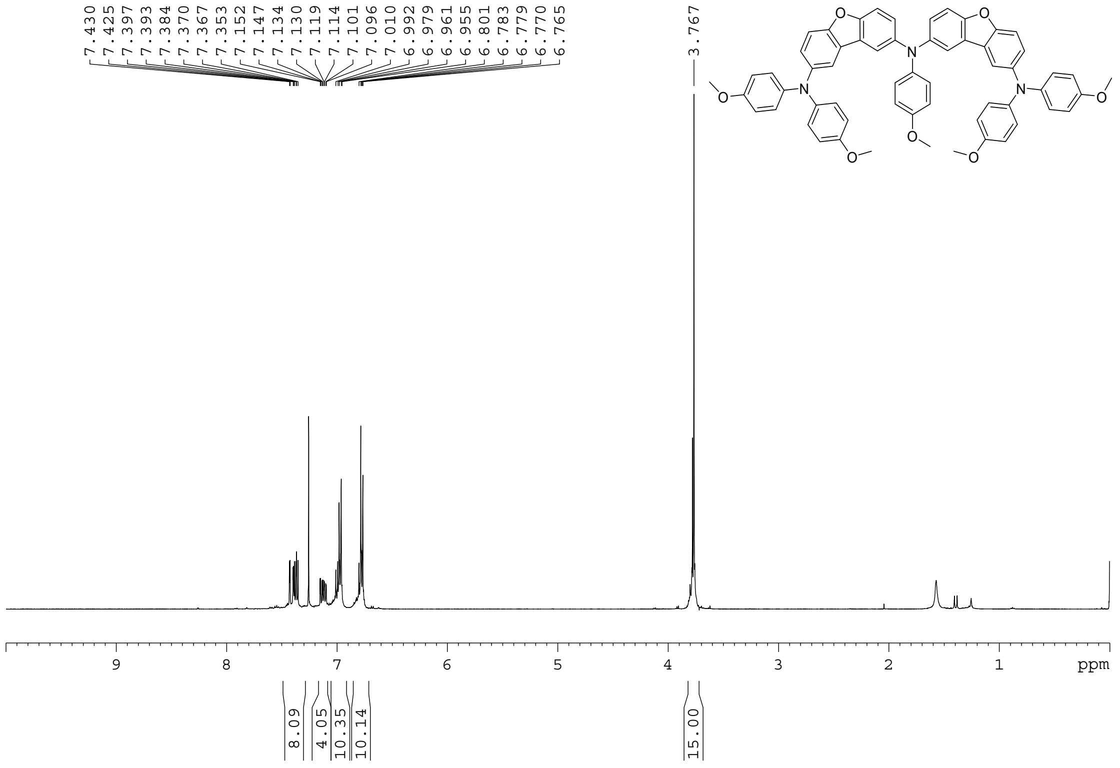

Figure. S8 ^{1}H NMR spectrum of **bDBF**.

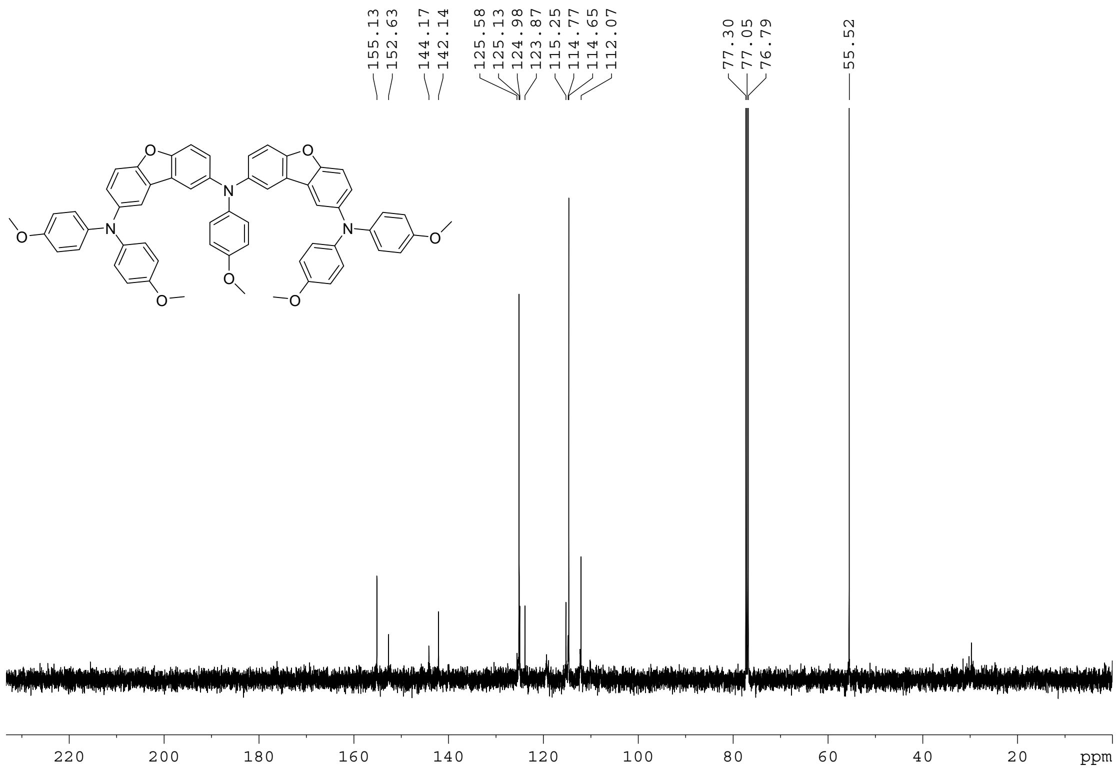

Figure. S9 ^{13}C NMR spectrum of **bDBF**.

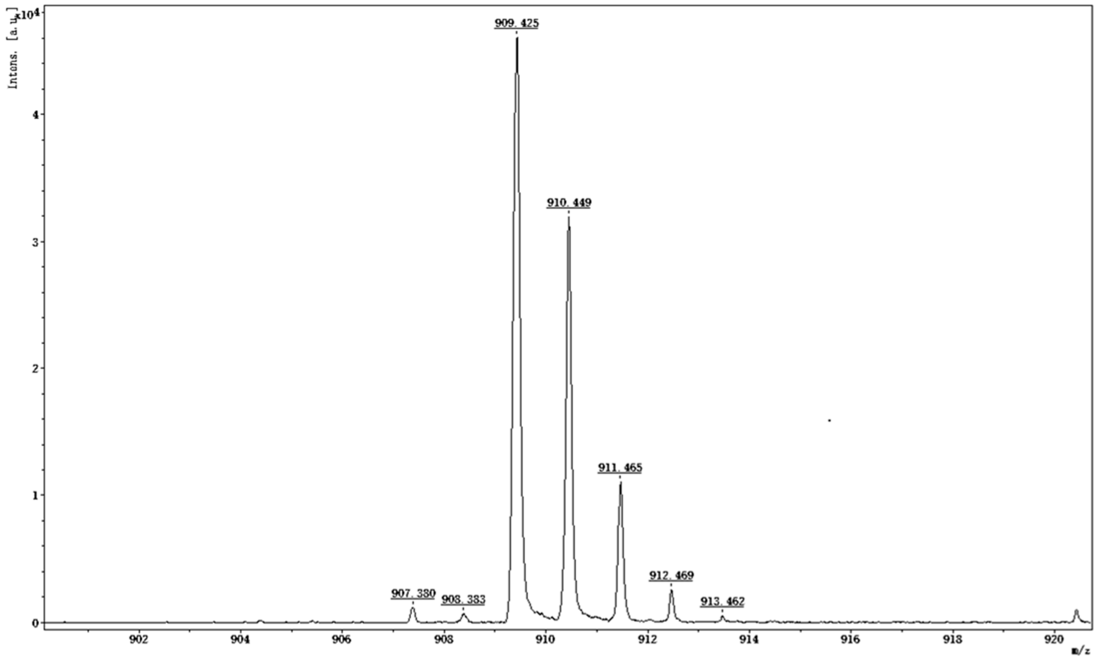

Figure. S10 MALDI-TOF mass spectrometry of **bDBF**.

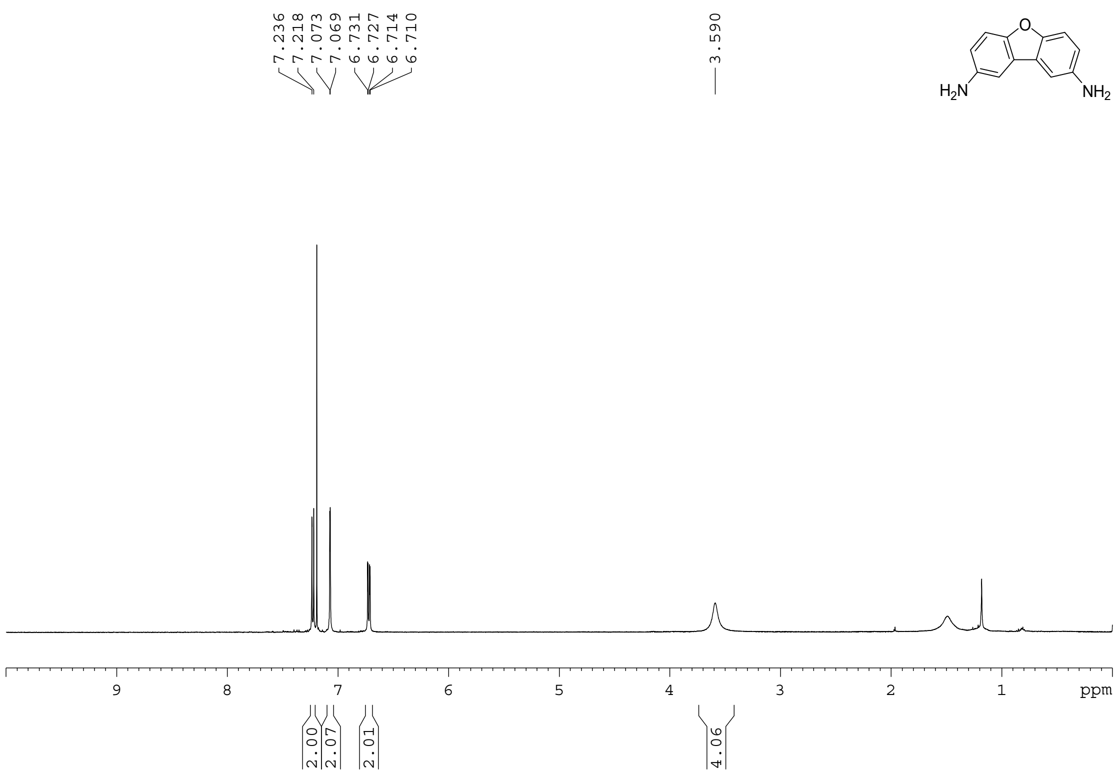

Figure. S11 ^{1}H NMR spectrum of **3**.

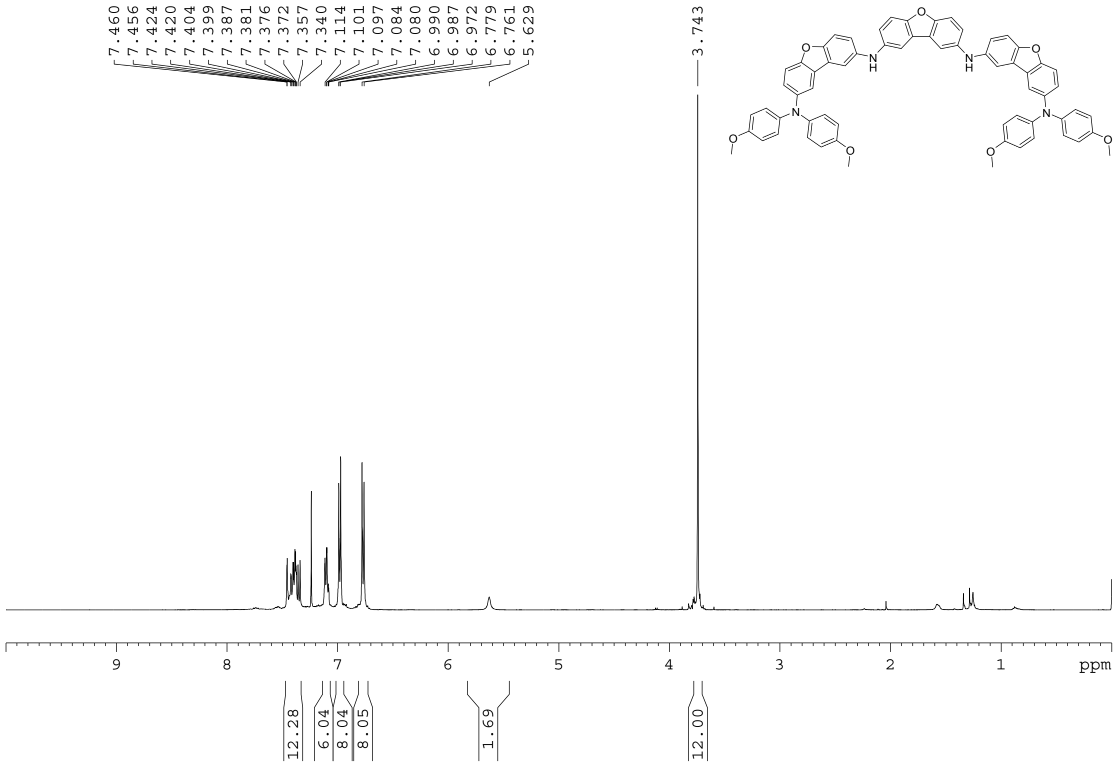

Figure. S12 ^{1}H NMR spectrum of **4**.

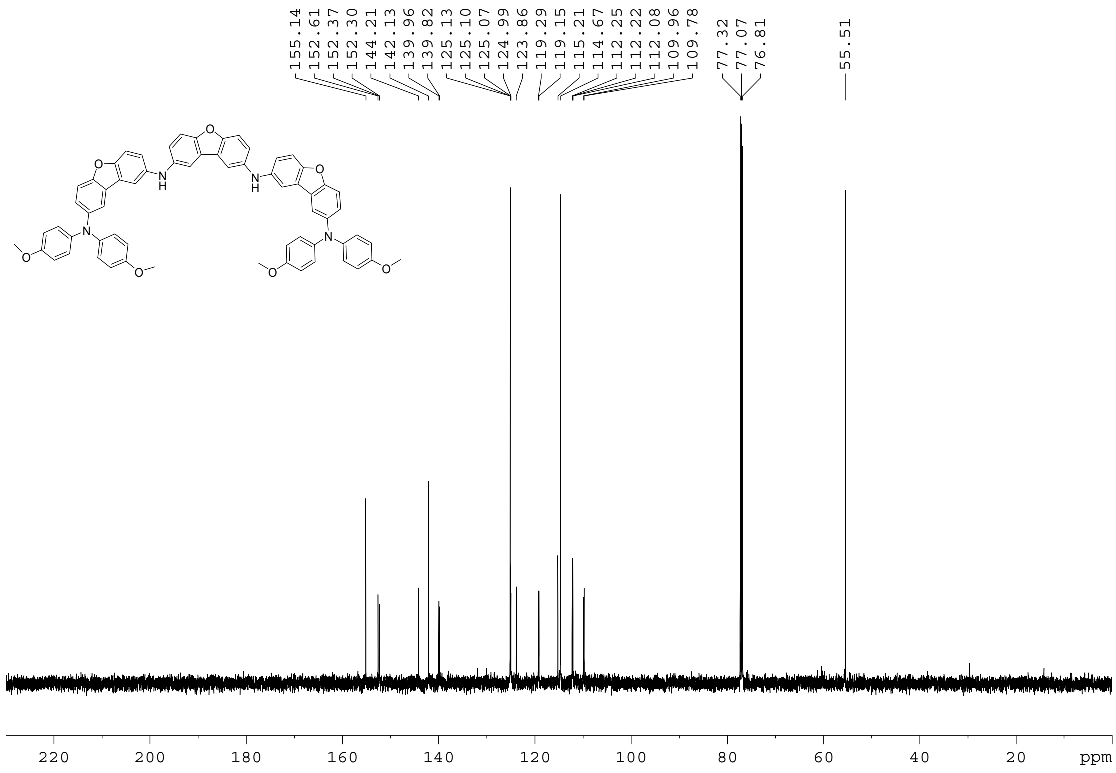

Figure. S13 ^{13}C NMR spectrum of **4**.

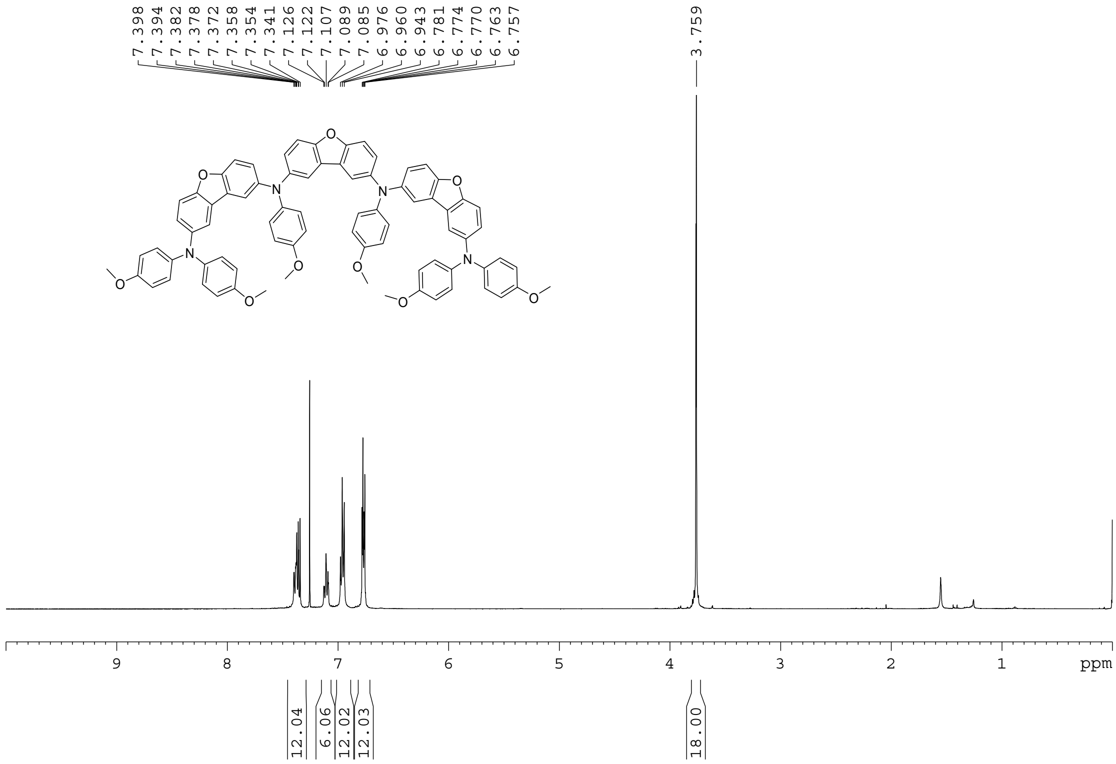

Figure. S14 ^{1}H NMR spectrum of **tDBF**.

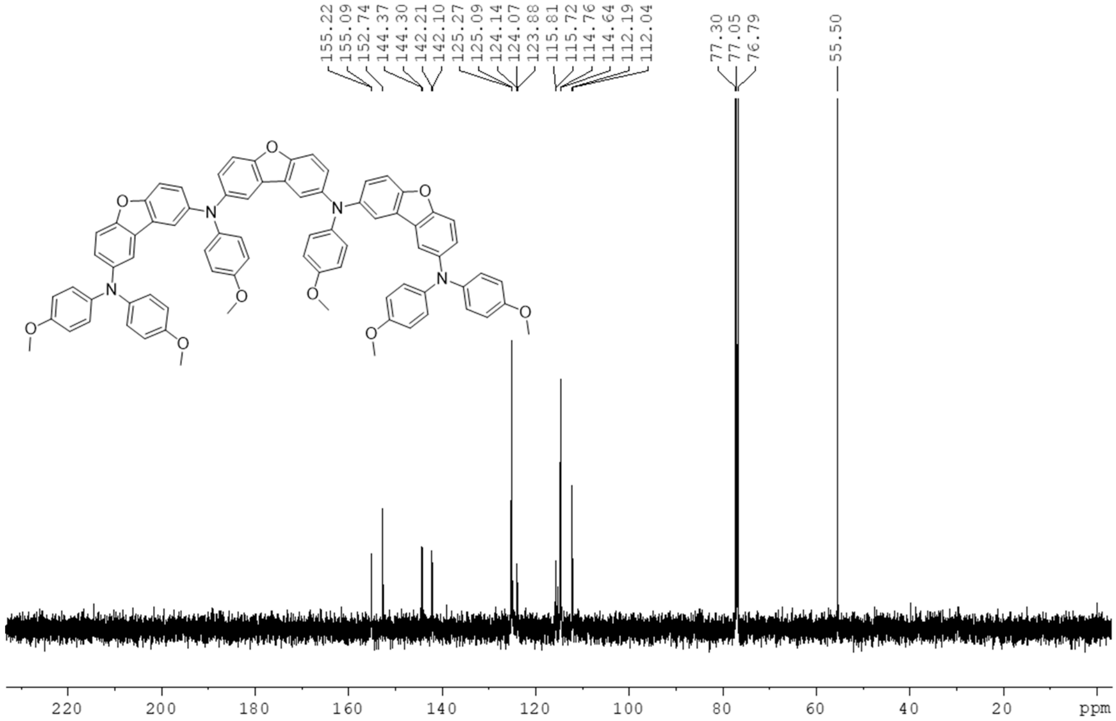

Figure. S15 ^{13}C NMR spectrum of **tDBF**.

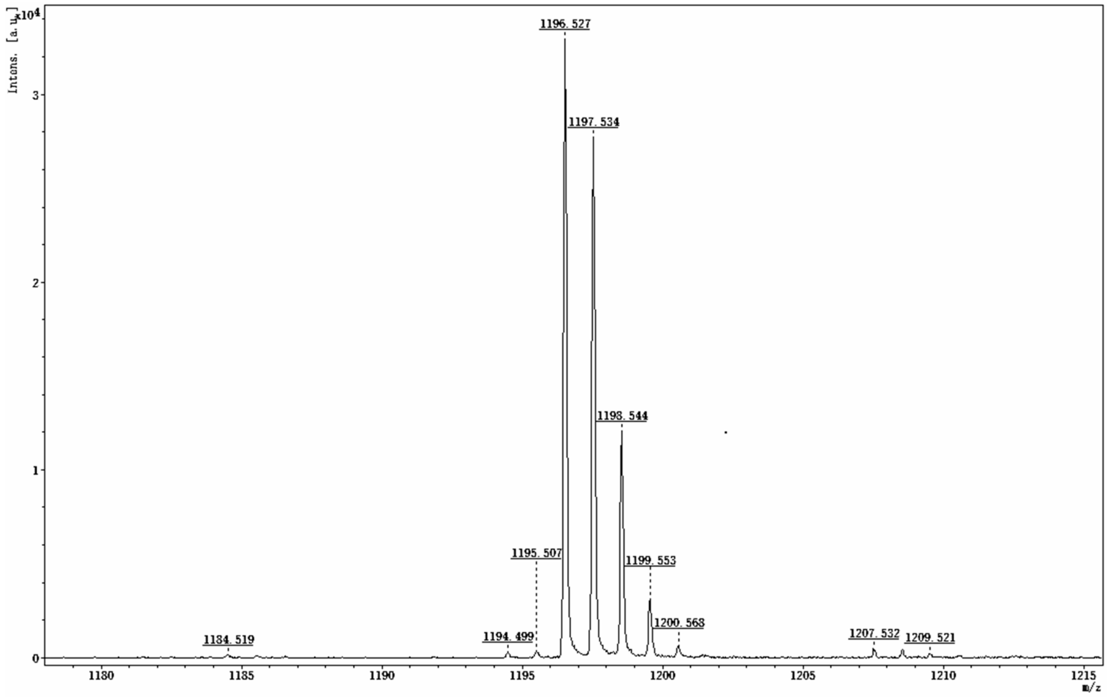

Figure. S16. MALDI-TOF mass spectrometry of **tDBF**.

2. UV-visible absorption

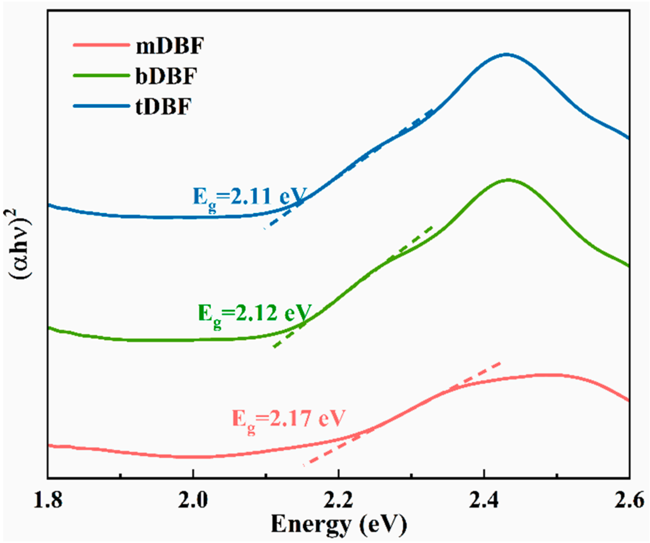

Figure. S17. Tauc curves from UV-visible absorption spectrum of the materials in the thin films.

3. The PSCs using different HTMs

Table S1 The relevant parameters obtained from PSCs with different HTMs

HTM	Device configuration	V_{oc} (V)	J_{sc} (mA cm^{-2})	FF (%)	PCE (%)	Ref.
P3HT	FTO/SnO_2/$(FAPbI_3)_{0.95}(MAPbBr_3)_{0.05}$ /Ga(acac)$_3$+P3HT/Au	1.15	25.50	83.80	24.60	[1]
CuPc	FTO/ns-TiO_2/c-TiO_2/ $(FAPbI_3)_{0.95}(MAPbBr_3)_{0.05}$/PMMA/CuPc/Au	1.08	24.87	79.29	21.25	[2]
CL-MCz	ITO/CL-MCz/ $(FA_{0.17}MA_{0.94}PbI_{3.11})_{0.95}(PbCl_2)_{0.05}$/$C_{60}$/BCP/Ag	1.17	24.15	84.60	23.90	[3]
MeO-2PACz	PEN(ITO)/MeO-2PACz/3F-2CN+ /$Cs_{0.05}(FA_{0.98}MA_{0.02})_{0.95}Pb(I_{0.98}Br_{0.02})_3$/$C_{60}$/BCP/Ag	1.14	25.36	83.57	24.08	[4]
P3CT	ITO/P3CT/ $(FA_{0.17}MA_{0.94}PbI_{3.11})_{0.95}(PbCl_2)_{0.05}$/$C_{60}$/ZrAcac/Ag	1.12	22.88	82.00	21.09	[5]
Spiro-OMeTAD	FTO/SnO_2/RACl+$FAPbI_3$/Spiro-OMeTAD/Au	1.18	25.69	86.15	26.08	[6]

4. Synthesis cost of HTMs

Table S2. Materials quantities and cost for the synthesis of 1-g mDBF, bDBF and tDBF.

Chemical name (purity)	Price of chemical (RMB/quantity)	Weight or volume of chemical			Material cost (RMB)		
		mDBF	bDBF	tDBF	mDBF	bDBF	tDBF
Dibenzo[*b, d*]furan (98%)	107.1/500 g	0.65 g	3.30 g	5.01 g	0.14	0.71	1.07
Bromine (≥99.5%)	239.9/500g	1.36 g	6.91	10.46 g	0.65	3.32	5.02
Sodium thiosulfate (AR)	18.8/500g	1.58 g	8.02 g	3.84 g	0.06	0.30	0.14
Bis-(4-methoxyphenyl)-amine (98%)	231.7/25g	1.00 g	1.15 g	1.47 g	9.27	10.66	13.62
p-Anisidine	27/25g		0.14 g			0.15	
Tri-tert-butylphosphine tetrafluoroborate (≥98%)	34.6/5 g	0.05 g	0.20 g	0.12 g	0.35	1.38	0.83
Tris(dibenzylideneacetone) dipalladium(0) (99.6%)	144.0/1 g	0.07 g	0.35 g	0.49 g	13.72	50.4	70.56
Cuprous iodide (Macklin (ML), 99.9%)	29.6/g			0.11 g			3.27
(S)-2,2'-bis(diphenylphosphino)-1,1'-binaphthyl (97%)	302.4/25 g			0.18 g			2.18
4,5-Bis(diphenylphosphino)-9,9-dimethylxanthene (98%)	30.8/5 g		0.20 g	0.26 g		1.23	1.60
N,N'-Dimethylethylenediamine (97%)	179.1/100 mL			0.15 mL			0.27
Sodium tert-butoxide (98%)	18/ 25 g	0.57g	1.51 g	2.13 g	0.41	1.09	1.53
Aqueous ammonia (AR)	12.5/500 mL			3.5 mL			0.09
Sodium sulfate anhydrous (AR)	15.2/500 g			5.21 g			0.16
Trichloromethane (AR)	43.2/500 mL	4 mL	20 mL	30 mL	0.35	1.73	2.60
Dichloromethane (AR)	220.0/25 L	302 mL	1.53 L	2.32 L	2.66	13.46	20.42
Toluene (AR)	200.0/25L	120 mL	1.13 L	1.72 L	0.96	9.04	13.76
Dimethyl sulfoxide (AR)	72.8/500 mL			2.5 mL			0.36
Petroleum ether (AR)	150.0/25 L	600 mL	2.31 L	3.12 L	3.60	13.86	18.72
Ethylacetate (AR)	225.0/25 L	308 mL	1.09 L	2.01 L	2.77	9.81	18.09
Column chromatography silica gel (200-300 mesh) (AR)	125.0/5kg	50 g	100 g	250 g	1.25	2.50	6.25
Total					**36.19 (5.11 US$/g)**	**119.64 (16.90 US$/g)**	**180.54(25.50 US$/g)**

5. Photovoltaic parameters of the devices

Table S3 Summary of photovoltaic parameters of n-i-p flexible PSCs employing different HTMs

HTM	V_{oc} (V)	J_{sc} (mA cm^{-2})	FF (%)	PCE$_{max}$ (%)
**Spiro-OMeTAD**	1.04	24.05	74.84	18.78
**mDBF**	0.65	6.11	66.16	2.65
**bDBF**	1.07	23.80	73.60	18.66
**tDBF**	1.08	23.54	76.47	19.46

Reference

1. Jeong, M. J.; Yeom, K. M.; Kim, S. J.; Jung, E. H.; Noh, J. H. Spontaneous interface engineering for dopant-free poly(3-hexylthiophene) perovskite solar cells with efficiency over 24%. Energy Environ. Sci. **2021**, *14*, 2419.
2. Kim, H.; Lee, K. S.; Paik, M. J.; Lee, D. Y.; Lee, S. U.; Choi, E.; Yun, J. S.; Seok, S. Il. Polymethyl methacrylate as an interlayer between the halide perovskite and copper phthalocyanine layers for stable and efficient perovskite solar cells. Adv. Funct. Mater. **2022**, *32*, 2110473.
3. Zhang, C. P.; Liao, Q. G.; Chen, J. Y.; Li, B. L.; Xu, C. Y.; Wei, K.; Du, G. Z.; Wang, Y.; Liu, D. C.; Deng, J. D.; Luo, Z. D.; Pang, S. P.; Yang, Y.; Li, J. R.; Li Yang, Guo, X. G.; Zhang, J. B. Thermally crosslinked hole conductor enables stable inverted perovskite solar cells with 23.9% efficiency. Adv. Mater. **2023**, *35*, 2209422.
4. Xie, L. S.; Du, S. Y.; Li, J.; Liu, C.; Pu, Z. W.; Tong, X. Y.; Liu, J.; Wang, Y. H.; Meng, Y. Y.; Yang, M. J.; Wei Li, W.; Ziyi Ge, Z. Y. Molecular dipole engineering-assisted strain release for mechanically robust flexible perovskite solar cells. Energy Environ. Sci. **2023**, *16*, 5423–5433.
5. Liao, Q.; Wang, Y.; Yao, X.; Su, M.; Li, B.; Sun, H.; Huang, J.; Guo, X. A. Dual-functional conjugated polymer as an efficient hole-transporting layer for high-performance inverted perovskite solar cells. ACS Appl. Mater. Interfaces **2021**, *13*, 16744–16753.
6. Park, J.; Kim, J.; Yun, H-S.; Paik, M. J.; Noh, E.; Mun, H. J.; Kim, M. G.; Shin, T. J.; Seok, S. Il. Controlled growth of perovskite layers with volatile alkylammonium chlorides. Nature **2023**, *616*, 724.
